# Effect of Cumulative Zinc Doses on Papillary Muscle Contractions and the Zinc Finger Protein ZEB1

**DOI:** 10.1007/s12011-025-04550-z

**Published:** 2025-02-18

**Authors:** Nı̇lufer Akgun-Unal, Aylı̇n Ustun, Sevban Bayirli, Omer Unal, Rası̇m Mogulkoc, Abdulkerim Kasım Baltacı

**Affiliations:** 1 Medical Faculty Department of Biophysics, Ondokuz Mayıs University, Samsun, Turkey; 2 Selcuk University Vocational School of Health Services, Konya, Turkey; 3 Istanbul Medipol University, Graduate School of Health Sciences Istanbul-Turkey, Istanbul, Turkey; 4 Medical Faculty Department of Physiology, Kirikkale University, Kirikkale, Turkey; 5 Medical Faculty Department of Physiology, Selcuk University, Konya, Turkey

**Keywords:** Zinc, ZEB1, Papillary muscle, Excitation–contraction coupling

## Abstract

In this study, we aimed to investigate the effects of cumulative doses of Zn^2+^ (by exposing samples to 1 µM, 10 µM, and 100 µM ZnCl_2_) on myocardial papillary muscle contractions isolated from rat hearts in vitro and the roles of the zinc finger protein ZEB1 in this effect. In these preparations, 100 µM ZnCl_2_ application in different protocols caused a decrease in contraction force and an increase in contraction time in both frequency-dependent parameters and pre-expected stimuli when compared to the control group. Our study data show that Ca^2+^ homeostasis is closely related to increasing Zn^2+^ doses (especially at 100 µM ZnCl_2_ dose). Secondly, the levels of ZEB1, a zinc finger protein, were also significantly lower in the 100 µM ZnCl_2_ group compared to the other groups, which seems to be related to the increase in Ca^2+^ that triggers ROS production at high doses of Zn^2+^. The data of our study, which we conducted to understand the Zn^2+^ concentrations in the heart and to reveal new mechanisms that play a role in the regulation of Ca^2+^ dynamics in heart tissue and is the first research in the literature on this subject, show that in vitro zinc application may have a dose-dependent effect on myocardial papillary muscle contractions.

## Introduction

Zinc (Zn^2+^), one of the most important trace elements in the body, is indispensable for the growth and development of microorganisms, plants, and animals [1]. Zn^2+^ is the only metal found in all enzyme classes and is a cofactor for more than 300 enzymes [2]. Zn^2+^ plays an important role as an extracellular and intracellular signaling factor, enabling communication between cells, transducing stimuli into intracellular responses, and regulating signaling pathways [3].

Total zinc in cells (up to 200 mM) is not very different from calcium; 30% is in the nucleus, 50% in the cytosol and organelles, and the rest in proteins [4]. In cardiomyocytes, the intracellular free Zn^2+^ concentration ([Zn^2+^]_i_) has been measured at less than 1 nM under physiological conditions [5], i.e., approximately 100 times less than the intracellular free Ca^2+^ concentration ([Ca^2+^]_i_) [6]. In addition, antioxidants cause an approximately 30-fold increase in [Zn^2+^]_i_ in cardiomyocytes while causing a twofold increase in [Ca^2+^]_i_ [7].

Cardiac excitation–contraction (EC) coupling is a process that governs cardiac contractility through the controlled release of Ca^2+^ from the sarcoplasmic reticulum (SR). In pathological conditions, channels become abnormally active and fail to remain closed during diastole, resulting in irregular contraction and electrical activity [8]. Deficient or excess Zn^2+^ intake has been shown to contribute to the pathology of some cardiomyopathies, including heart failure [8]. Cardiomyocytes contain a measurable free Zn^2+^ pool of 100 pM in the cytosol [9]. This is particularly important given studies showing that zinc increases to 50 nM during Zn^2+^-signaling events, as changes in Zn^2+^ concentration will have a significant impact on Ca^2+^ homeostasis [10]. Extracellular Zn^2+^ has also been shown to enter cardiomyocytes via L-type Ca^2+^ channels, similar to Ca^2+^ [11]. These Zn^2+^ fluctuations help regulate Ca^2+^ homeostasis, highlighting a potential role for Zn^2+^ in fine-tuning Ca^2+^ release events to control the force and duration of cardiac contractions.

Under pathophysiological conditions, [Zn^2+^]_i_ is reported to be elevated and reach levels in the high nM range (approximately 30 nM) [8]. Under these conditions is uncoupled from the regulatory effects of cytosolic Ca^2+^ and comes under the control of Zn^2+^. This may lead to ion channel activity, which is associated with heart failure and fatal arrhythmias. This is a result of impaired EC connectivity as a result of altered Ca^2+^ homeostasis function. The effects of zinc on contractile force and cardiac calcium release are not fully understood.

Oxidative stress plays an important role in the pathogenesis of many important diseases, including cardiovascular system diseases [12]. However, it is now accepted that antioxidants are also produced in healthy tissues and serve as signaling molecules [13]. Therefore, maintaining the reductive/oxidative balance within the cell is critical for many cellular functions, including cardiomyocytes, and failure to maintain this balance contributes to cardiac dysfunction [14, 15]. Myocardial oxidative stress, which can be beneficial at low doses, can play a pathophysiological role by causing excessive production of ROS-induced ROS release [14, 16]. In the cytosol of cardiomyocytes, excessively increased levels of zinc lead to an acute increase in ROS, acting as a second messenger of extracellular signals similar to Ca^2+^ [17]. ROS activates epithelial-mesenchymal transition (EMT) in pathological conditions, especially in cancer cells [18, 19]. EMT is regulated by a small set of transcription factors, including ZEB1, a member of the ZEB family [20, 21]. ZEB1 transcription factors have been shown to activate the adaptive antioxidant response in skeletal muscle [22]. These findings suggest that ZEB factors may serve as potential therapeutic targets to modulate the antioxidant response in pathophysiological conditions.

Based on the above evidence and based on the multiple functions of Zn^2+^, we can assume that Zn^2+^ has an important role in modulating excitation–contraction coupling under physiological conditions, and pathological processes occur in cases of zinc deficiency or excess. However, little is known about the dose-dependent mechanisms of Zn^2+^ during myocardial papillary muscle contraction or its changes during the cardiac cycle. Therefore, we aimed to investigate a dose-dependent effect of cumulative Zn^2+^ doses on myocardial papillary muscle contractions in vitro in order to understand the Zn^2+^ concentrations in the heart and to reveal new mechanisms involved in the regulation of Ca^2+^ dynamics in cardiac tissue and to determine the potential role of ZEB1 on cardiac contractions.

## Materials and Methods

### Ethical Statement

This study was conducted with approval from the Selçuk University Experimental Medicine Research and Application Center Animal Experiments Ethics Committee with decision number 2023–27.

### Animal Model and Study Design

This study was conducted on 20 female Wistar albino rats (6 months old), weighing 300–350 g, obtained from Selcuk University Experimental Medicine Research and Application Center. Animals were handled with all precautions necessary to avoid suffering in accordance with the ARRIVE guidelines. All animals were exposed to a 12-h light–dark cycle and had free access to tap water. They were fed with standard chow daily ad libitum. In the study where a total of 20 rats were used, the experimental animals were divided into 4 groups of equal numbers.

Control group (C): Myocardial papillary muscle contraction recordings were taken from the animals in this group without administering zinc into the Krebs solution.

1 micromol/L ZnCI_2_ group (1 µM ZnCI_2_): Myocardial papillary muscle contraction recordings were taken by administering 1 micromol/L ZnCI_2_ into the Krebs solution.

10 micromol/L ZnCI_2_ group (10 µM ZnCI_2_): Myocardial papillary muscle contraction recordings were taken by administering 10 micromol/L ZnCI_2_ into the Krebs solution.

100 micromol/L ZnCI_2_ group (100 µM ZnCI_2_): Myocardial papillary muscle contraction recordings were taken by administering 100 micromol/L ZnCI_2_ into the Krebs solution.

### Papillary Muscle Contraction Activity Measurement

Papillary muscles isolated from left ventricles were tied with 6/0 silk thread at both ends. Tissues prepared in this way were taken to a 30 ml volume isolated organ bath (MAY IOBS99 Isolated Tissue Bath and Circulator, Commat Ltd.) containing fresh Krebs solution (135 mM NaCl, 5 mM KCl, 2.5 mM CaCl_2_, 1 mM MgSO_4_.7H_2_O, 1 mM NaH_2_PO_4_.2H_2_O, 15 mM NaHCO_3_, 11 mM glucose). The temperature of the Krebs solution was kept constant at 33 °C by passing through the heat jacket in the organ bath (MAY WBC 3044 Water Bath and Circulator, Commat Ltd.) [23]. One of the metal hooks on the strings to which the papillary muscles are attached was attached to a manually controlled micromanipulator, and the other was attached to a force transducer (FDT05 Force Displacement Transducer, Grass Co.), and the tension of the muscle was adjusted to see maximum contraction. Supramaximal square-shaped stimuli were given using a stimulator (MAY ISO 150-C Stimulus Isolation Power Supply) in the form of a field stimulus through an electrode designed to remain between the anode–cathode ends of the papillary muscle. All data related to the contraction were collected on a hard disk at a sampling rate of 1 kHz via an analog–digital converter (MP36 four channel data acquisition unit, Biopac System Inc.) and its own software (BSL PRO 3.7.5, Biopac System Inc.). After the completion of the contraction recordings, the papillary muscles were removed from the isolated organ bath, the ropes attached to their ends were untied, and their dry weights were measured with a scale with 0.1 mg sensitivity (Ohaus PA2224C) [24].

The Bowditch effect describes the relationship between the contraction force and the contraction velocity in terms of force-frequency [25]. An investigation into frequency-dependent responses was conducted to understand this relationship. Research into the frequency-dependent contraction parameters of the papillary muscle found that a frequency of 0.2 Hz was not associated with muscle fatigue. Examination of the frequency increase steps revealed that the difference compared to the control group diminished further from low frequency to 5 Hz, and this disparity ceased to be statistically significant at 5 Hz [13]. Furthermore, research has established a direct association between the rate of muscle contraction and the force generated. A correlation was found between decreased contraction rate and lower contractility in the failing heart muscle. The standard pacing frequency was lowered to 0.2 Hz and then gradually increased to 5 Hz, following a progression of specific frequencies (0.2, 0.5, 1, 2, 3, 4, and 5 Hz) [26, 27].

After the papillary muscle was placed in the organ bath, square-shaped stimuli of 10–15 V (supramaximal) and 2 ms duration were given at a basic frequency of 0.2 Hz. At this stage, the Krebs solution was changed every 15 min for a total of 1 h to ensure that the tissue was as close to physiological conditions as possible. During this time, contractions were monitored simultaneously, and it was observed that the contraction amplitudes became stable at the end of the 1-h period. Then, to see the frequency-dependent contraction responses, square-shaped stimuli of 10–15 V (supramaximal) and 2 ms duration were given at frequencies of 0.2, 0.5, 1, 2, 3, 4, and 5 Hz, respectively, and starting from the lowest frequency, 20 peak values were counted for each frequency and then the contraction curves were recorded by moving to the next higher frequency [28, 29]. After the recording was completed, the solution was refreshed and kept at the basic stimulus frequency for 15 min.

In the other protocol, responses to pre-waited stimuli were recorded. In order to investigate the mechanisms of Ca^2+^ uptake-release from the SR, 10, 20, 30, 40, 50, 60, and 70 s were waited between every 100 s of recording with 10–15 V, 2 ms duration square-shaped stimuli at 0.2 Hz frequency, and the parameters obtained as a result of this protocol were calculated from the first contraction curves recorded after the waiting period [28, 30].

### ELISA Analysis

A commercial ELISA kit (Bioassay Technology/BT Laboratory, China) with catalog number E0908Ra was used to determine the ZEB1 protein levels of papillary muscles taken from experimental animals by the Elisa method. For standardization, tissue weights were weighed and recorded using a precision balance. The tissue was broken down and homogenized in Misonix’s microscanultrasonic tissue disintegrator at a level of 10% homogenate (tissue weight (g):PBS (ml) volume = 1:9) in PBS (pH 7.4) and at 4 °C. The homogenates formed were centrifuged at 5000 × g for 5 min, and the supernatants were transferred to clean tubes. In order to reach room temperature, the reagents and samples were kept at room temperature, and classic and sample wells were determined on the plate. Then, 50 µl of classic solutions containing biotinylated rat ZEB1 antibody was added to the identified classic wells. Samples were added to the sample wells in the specified order as 40 µl, and 10 µl of biotinylated ZEB1 antibody was added. Then, 50 µl of streptavidin-HRP was added to these classic and sample wells and mixed completely. The plate was covered. It was incubated at 37 °C for 60 min. The plate sealer was removed, and the washing process was performed. Then, the liquids in the wells were aspirated and soaked with 300 µl of washing solution for 30–60 s 5 times. After washing 5 times, the liquids were aspirated. After the washing process, the first 50 µl of substrate solution A and then 50 µl of substrate solution B were added to each well (classic and sample). The plate was closed so that it would be clean, and the plate was incubated at 37 °C for 10 min in the dark to allow the reaction to occur and obtain a blue color. When the incubation period was over, the sealer was removed, and 50 µl of stop solution was added to each well. Therefore, the reaction was terminated. It was observed that the blue color had turned yellow. Then, the plate was read in 10 min on a microplate reader set to 450 nm wavelength, and the absorbance values were determined. Sample concentrations were calculated from the provided standard curve. The amounts of the samples were determined in ng/ml.

### Statistical Analysis

Statistical tests were performed using the GraphPad Prism 8 program. One-way ANOVA was used to compare the means of the data belonging to the groups regarding contraction and ZEB1 levels in a single direction. In addition, Tukey post hoc test was used to determine which groups the difference was between. Differences at the level of *p* < 0.05 were accepted as significant.

## Results

**Fig. 1:**
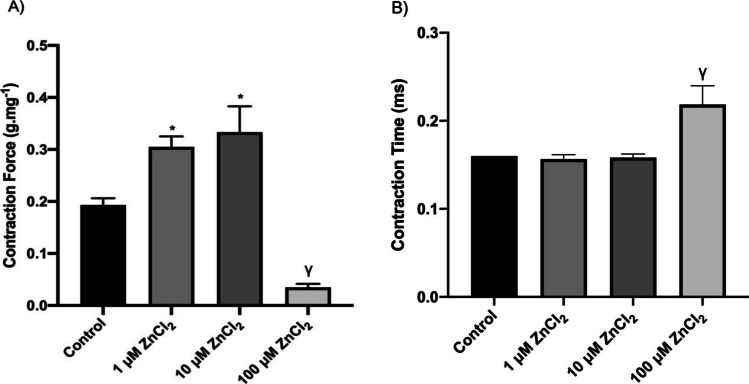
Recording parameters of all subjects with stimuli at 0.2 Hz frequency: **A** contraction force (CF) (g.mg^−1^) and **B** contraction time (CT) (ms). Response values to stimuli at 0.2 Hz frequency are given as mean ± standard error. * indicates significance between C and 1 and 10 µM ZnCI_2_ groups; γ indicates significance between C and 100 µM ZnCI_2_ groups (*p* < 0.05)

Contraction force (CF) and contraction time (CT) values of contraction records taken from experimental groups with 0.2 Hz frequency stimuli accepted as the basic parameter in myocardial papillary muscle are shown in Fig. 1. As can be seen from Fig. 1a and b, 100 µM ZnCI_2_ application caused a significant decrease in CF parameter and a significant prolongation in CT parameter between in vitro ZnCI_2_ applied and not applied groups (*p* < 0.05). Also, although there was a significant increase in CF parameter between 1 and 10 µM ZnCI_2_ groups and control group from cumulative ZnCI_2_ doses (Fig. 1a, *p* < 0.05), there was no significant difference in CT parameter (Fig. 1b).

**Fig. 2:**
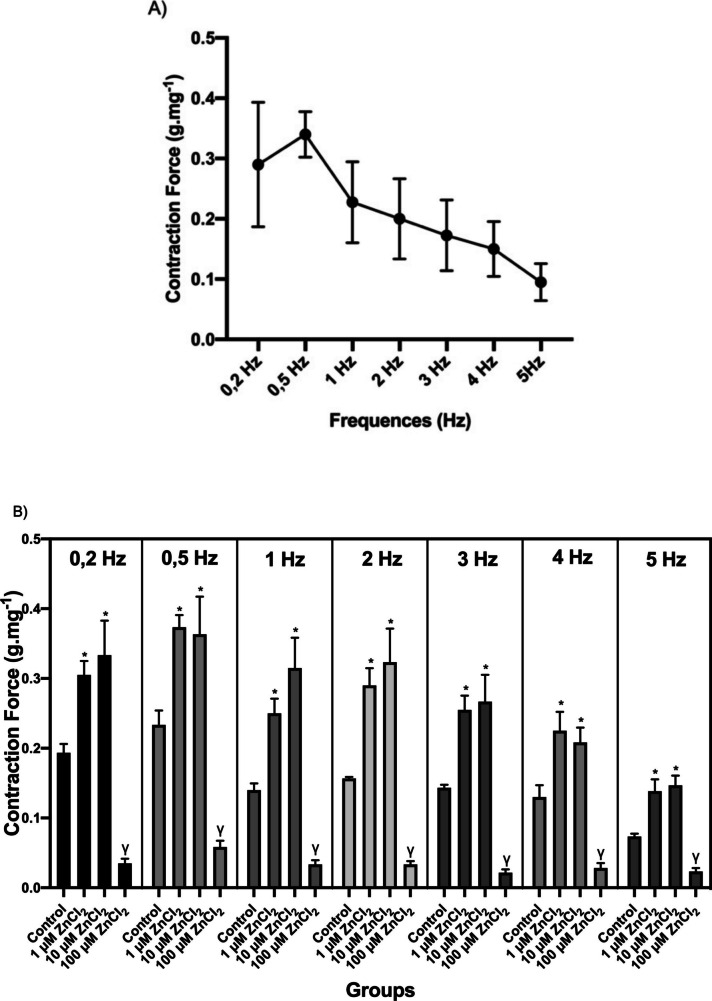

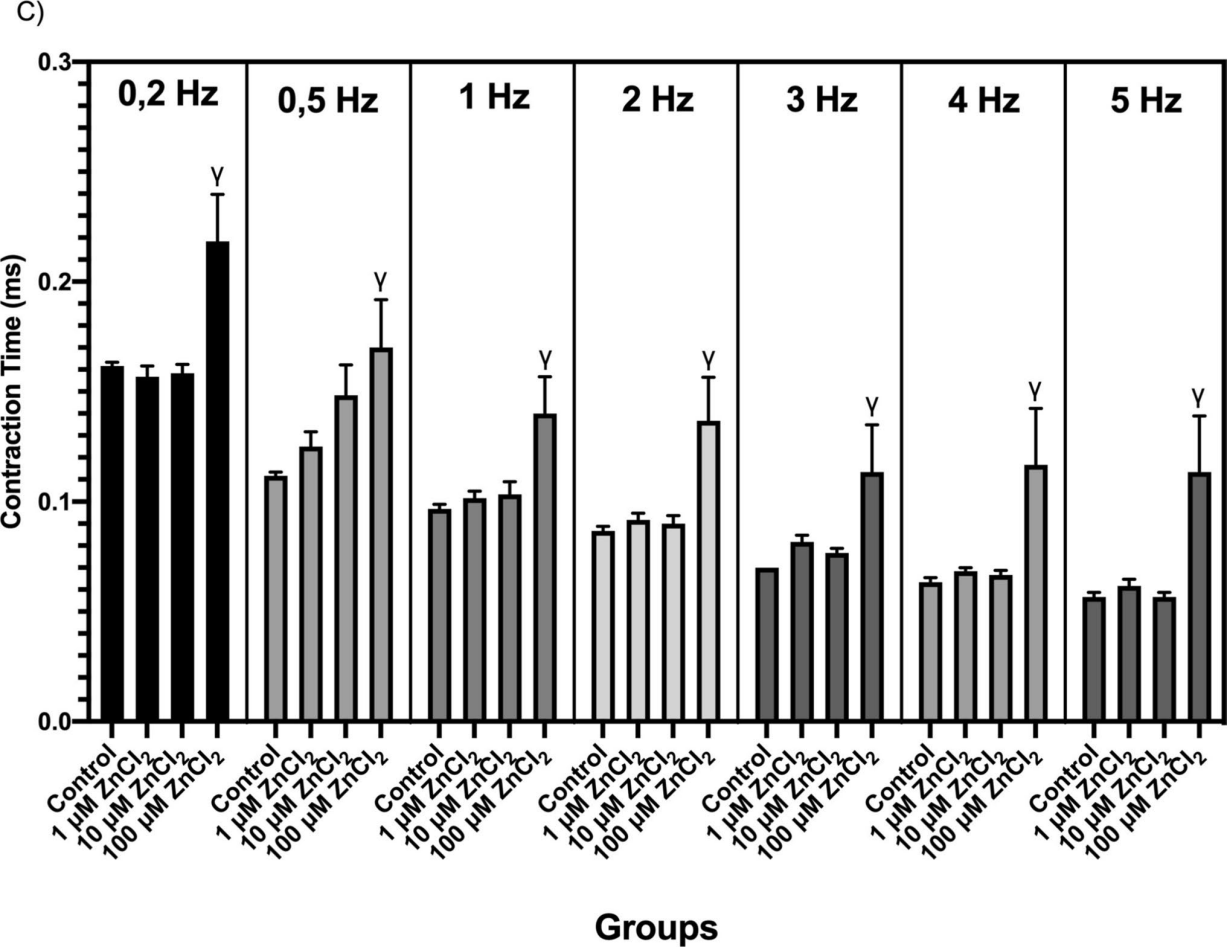
CF and CT values of the stimulus frequency-contraction relationship. **A, B** Contraction force (CF) (g.mg^−1^) and **C** contraction time (CT) (ms) recording values. Contraction force (CF) and contraction time (CT) response values to stimuli at different frequencies are given as mean ± standard error. * indicates significance between C and 1 and 10 µM ZnCI_2_ groups; γ indicates significance between C and 100 µM ZnCI_2_ groups (*p* < 0.05)

In order to determine the responses of isolated rat myocardial papillary muscles to stimuli at different frequencies (0.2, 0.5, 1, 2, 3, 4, and 5 Hz), contraction curves were recorded, and CF and CT parameters from the relevant curves are given in Fig. 2. In the groups to which ZnCI_2_ was added, there was a decrease in the CF induced in papillary muscle strips by electrical stimulation under isometric conditions in the frequency range of 0.5–5 Hz (Fig. 2a). We also determined whether cumulative ZnCI_2_ exposure had an effect on different frequencies. As can be seen in Fig. 2b, 100 µM ZnCI_2_ caused a significant decrease in CF (*p* < 0.05), while 1 and 10 µM ZnCI_2_ groups showed an increase in all frequencies compared to the control group (*p* < 0.05). In addition, our analysis of CT parameters revealed significant differences in the 100 µM ZnCI_2_ group (Fig. 2c). As can be seen from the graph in Fig. 2c, CT was significantly prolonged in the 100 µM ZnCI_2_ group compared to the control group (*p* < 0.05).

**Fig. 3:**
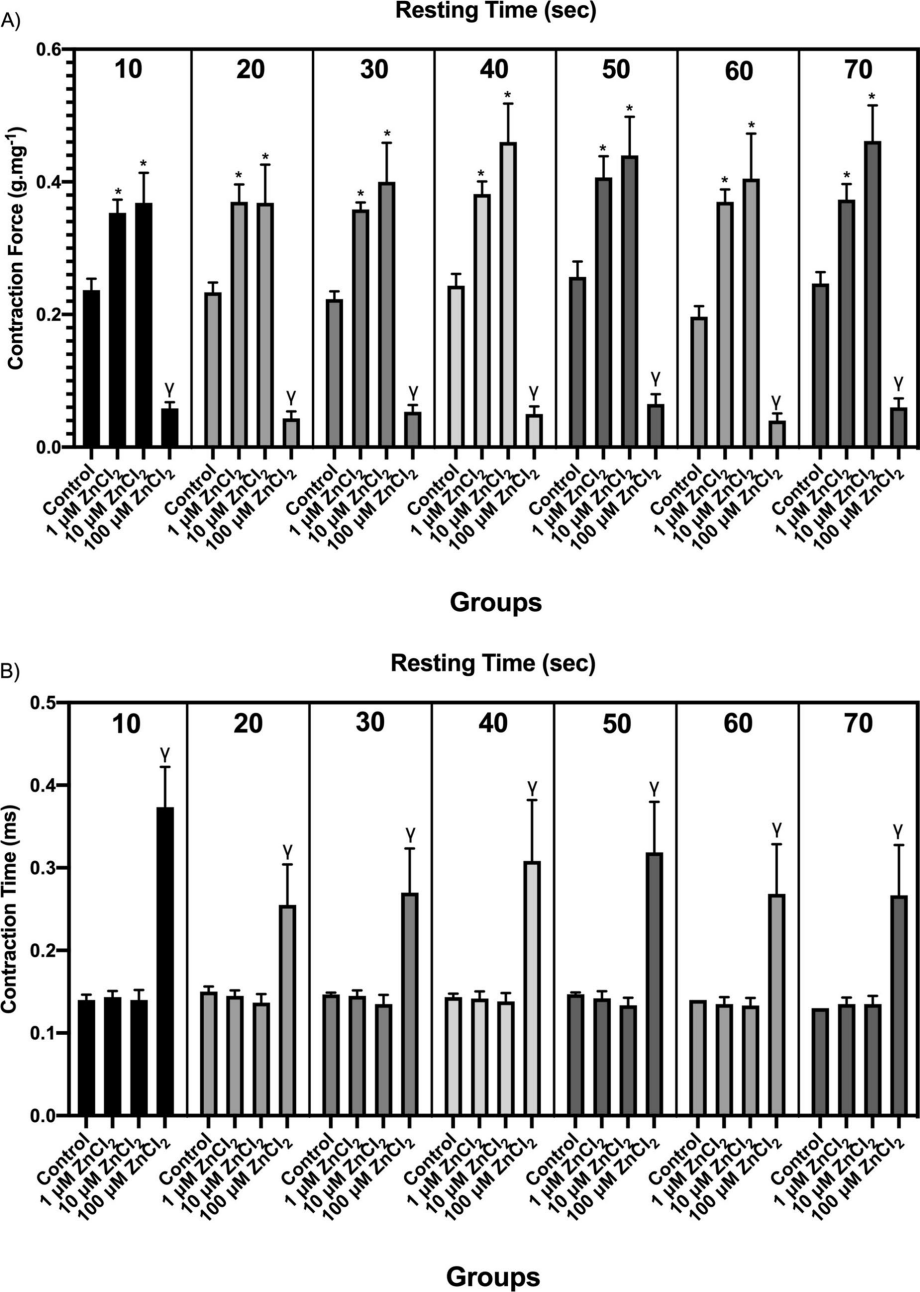
Recording parameters of the pre-existing stimulus-contraction relationship: **A** contraction force (CF) (g.mg^−1^) and **B** contraction time (CT) (ms). Contraction force (CF) and contraction time (CT) response values to pre-existing stimuli are given as mean ± standard error. * indicates significance between control and 1 and 10 µM ZnCI2 groups; γ indicates significance between control and 100 µM ZnCI2 groups (*p* < 0.05)

In order to investigate the mechanisms of Ca^2+^ uptake-release from SR, CF and CT values obtained by waiting for 10, 20, 30, 40, 50, 60, and 70 s between every 100-s recordings with 10–15 V, 2 ms duration square-shaped stimuli at 0.2 Hz frequency are shown in Fig. 3. While a significant decrease was observed in CF values during the 70-s waiting period with 100 µM ZnCI_2_ compared to the control group (*p* < 0.05), a significant increase was determined in the 1 and 10 µM ZnCI_2_ groups (*p* < 0.05, Fig. 3a). In the recordings taken with pre-waited stimuli, 100 µM ZnCl_2_ caused a prolongation in CT values for all durations (*p* < 0.05). In addition, no significant difference was found between the 1 and 10 µM cumulative ZnCl_2_ groups and the control group during the 70-s waiting period (Fig. 3b).

**Fig. 4:**
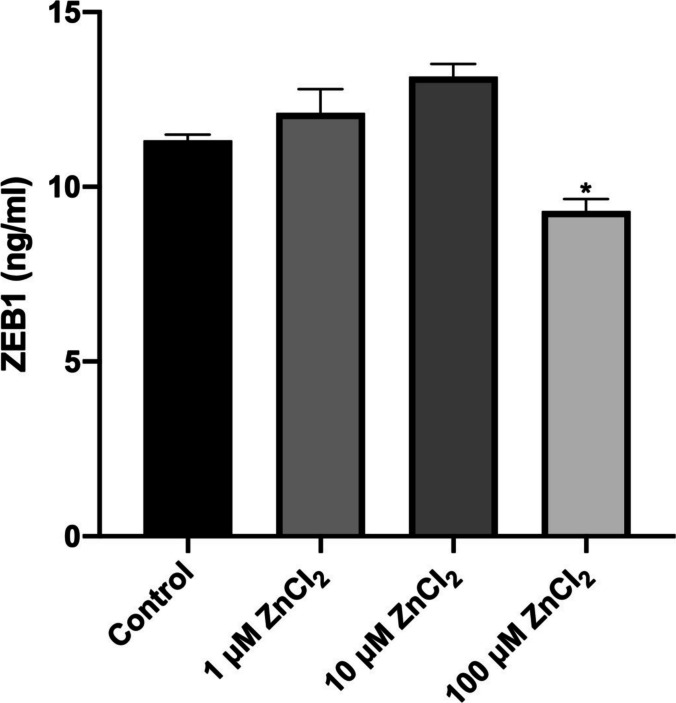
Papillary muscle ZEB 1 Elisa values (ng/ml). ZEB1 gene expression values are given as mean ± standard error. * indicates the degree of significance between groups at the *p* < 0.05 level

The cumulative in vitro ZnCI_2_ application-induced papillary muscle ZEB1 levels in rats treated with 1 µM, 10 µM, and 100 µM ZnCI_2_ are given in Fig. 4. It was determined that ZEB1 values were significantly decreased in the 100 µM ZnCI_2_ group compared to the other groups (*p* < 0.05). The ZEB1 levels of the control, 1 µM, 10 µM, and 100 µM ZnCI_2_ groups were measured as 11.33 ± 0.37 ng/ml, 12.11 ± 1.69 ng/ml, 13.15 ± 0.88 ng/ml, and 9.31 ± 0.86 ng/ml, respectively, and it was determined that the 100 µM ZnCI_2_ group had a statistically significant decrease in ZEB1 value (mean ± SD). However, although there was a numerical increase in ZEB1 values in the 1 µM and 10 µM ZnCI_2_ groups compared to the control group, no statistically significant difference was observed (12.11 ± 1.69 ng/ml, 13.15 ± 0.88 ng/ml, mean ± SD, respectively) (*p* > 0.05).

## Discussion

Basically, our current data first showed that 100 µM in vitro Zn^2+^ concentrations caused a significant prolongation of contraction duration, a significant decrease in CF, and a slowdown in Ca^2+^ uptake and release mechanisms from the SR in papillary muscle contraction parameters in the rat heart. Again, our current data secondly showed that ZEB1, a zinc-binding finger protein, was significantly reduced in papillary muscle ZEB1 levels with 100 µM in vitro zinc administration. Zn^2+^ plays an important role in cardiac function, including excitation–contraction coupling [5, 17, 31, 32], excitation-transcription coupling [11], and cardiac ventricular morphogenesis [33]. In the heart, zinc regulation is tightly regulated to maintain labile Zn^2+^ concentrations, although [Zn^2+^]_i_ is low. In a study, it was reported that the total extracellular Zn^2+^ concentration value ranged from 10 μM to high μM concentrations, but the total [Zn^2+^]_i_ value was around 200 μM [34]. If the exchangeable Zn^2+^ concentration falls outside a narrow range, this causes cardiac dysfunction, including altered contractile strength [7, 35]. This situation emphasizes the importance of Zn^2+^ homeostasis in cardiovascular functioning. Therefore, when our current data are combined with literature data, the increase in CF after 1 and 10 µM in vitro Zn^2+^ application and the prolongation of contraction duration after 100 µM in vitro Zn^2+^ application can be summarized as Zn^2+^ release during the cardiac cycle via impaired Ca^2+^ homeostasis may be due to the increase in intracellular free Ca^2+^.

The second important observation in this study is that the ZEB1 gene, a zinc transport protein, may be a potential target in cardiomyocytes for the proliferation and invasion of various cells [36, 37]. ZEB factors have been identified as potential therapeutic targets to modulate the adaptive antioxidant response in pathophysiological conditions and diseases caused by redox imbalance [36, 37].

Studies show that Zn^2+^ release during the cardiac cycle is due to the ionic exchange on these proteins, as well as the increase in intracellular free Ca^2+^, which triggers ROS production, which mostly causes changes in the metal binding properties of metallothionein and other redox-active proteins [17, 38]. It is known that after the formation of heart failure and other arrhythmic diseases, the gene expression of ZEB1 is significantly induced in studies conducted in mice [39]. Since [Zn^2+^]_i_ is extremely low in cardiac cells, there is increasing interest in studies conducted to understand the role of free Zn^2+^ in cell survival and the related molecular mechanism [5]. In our study, in line with the above literature data, the Ca^2+^ increase significantly decreased papillary muscle ZEB1 levels as a result of 100 µM in vitro Zn^2+^ application.

Our data demonstrate the ability of Zn^2+^ to directly activate Ca^2+^ homeostasis at 1 nM concentration and that it has a much higher affinity for Zn^2+^ than for Ca^2+^ (approximately 3 orders of magnitude). These data suggest that Ca^2+^ homeostasis is closely related to intracellular Zn^2+^ levels. Woodier et al. [32] also showed that Zn^2+^ modulates both the frequency and amplitude of Ca^2+^ waves in cardiomyocytes in a concentration-dependent manner. In our study, lowering [Ca^2+^]i to subactivating concentrations did not eliminate Ca^2+^ waves in the presence of 1 nM Zn^2+^. These data suggest that channel dysregulation via Zn^2+^ dyshomeostasis may play a fundamental role in the development of heart failure and other arrhythmic diseases.

## Limitations

- Importantly, although we evaluated the results of the post-rest potentiation protocol we applied during mechanical activity recordings in this study on SR and intracellular calcium levels, further studies are needed to provide a deep understanding of the intracellular calcium levels and the molecular mechanisms associated with them. Nevertheless, despite this limitation, our study provides valuable evidence regarding the important effects of Zn^2+^ on the regulation of Ca^2+^ dynamics in cardiac tissue and highlights the potential for further research in this area.
- The study was applied on female rats since it was not hypothesized that sex-dependent differences would be revealed.
- In our study, no parameters were considered as markers of oxidative stress. This is another limitation of our study.

## Conclusion

In conclusion, we have shown in this study that any pathological stimulus can cause changes in cardiac contractile parameters, which can either alter ZEB1 levels or Ca^2+^ release from the SR, or both, leading to significant decreases and increases in contractile force and duration, respectively. Furthermore, these new data provide an explanation linking Zn^2+^ dyshomeostasis to certain cardiomyopathies characterized by defective contractility and dysregulated Ca^2+^ responses. However, in order to validate a model for integrated Zn^2+^ signaling in the heart, a more detailed understanding of the molecular mechanism by which Zn^2+^ modulates its function on Ca^2+^ homeostasis and its further effects on cardiac function is needed. Determining the origin of Zn^2+^ levels during the cardiac contraction-relaxation cycle in both health and disease will also be crucial to advance our understanding. Understanding Zn^2+^ concentrations in the heart and uncovering new mechanisms involved in regulating Ca^2+^ dynamics in cardiac tissue may highlight potential new drug targets to combat heart failure, cardiomyopathy, and lethal arrhythmias.

## Data Availability

Research Data Policy and Data Availability Statement The datasets generated during and/or analysed during the current study are available from the corresponding author on reasonable request.
